# Caffeic Acid-PLGA Conjugate to Design Protein Drug Delivery Systems Stable to Irradiation

**DOI:** 10.3390/jfb6010001

**Published:** 2015-01-05

**Authors:** Francesca Selmin, Francesco Puoci, Ortensia I. Parisi, Silvia Franzé, Umberto M. Musazzi, Francesco Cilurzo

**Affiliations:** 1 Università degli Studi di Milano, Department of Pharmaceutical Sciences—via Giuseppe Colombo, 71-20133 Milano, Italy; E-Mails: francesca.selmin@unimi.it (F.S.); silvia.franze@unimi.it (S.F.); umberto.musazzi@unimi.it (U.M.M.); 2 Università degli Studi della Calabria, Department of Pharmacy, Health and Nutritional Sciences, 87036 Arcavacata di Rende, Italy; E-Mails: francesco.puoci@unical.it (F.P.); ortensia.parisi@unical.it (O.I.P.)

**Keywords:** anti-oxidant, microencapsulation, drug release, caffeic acid, grafting, γ-irradiation, microspheres, ovalbumin, poly(lactide-co-glycolide), sterilization

## Abstract

This work reports the feasibility of caffeic acid grafted PLGA (g-CA-PLGA) to design biodegradable sterile microspheres for the delivery of proteins. Ovalbumin (OVA) was selected as model compound because of its sensitiveness of γ-radiation. The adopted grafting procedure allowed us to obtain a material with good free radical scavenging properties, without a significant modification of *M_w_* and *T_g_* of the starting PLGA (*M_w _*_PLGA_ = 26.3 ± 1.3 kDa *vs.* *M_w _*_g-CA-PLGA_ = 22.8 ± 0.7 kDa; *T_g_*_ PLGA_ = 47.7 ± 0.8 °C *vs.* *T_g_*_ g-CA-PLGA_ = 47.4 ± 0.2 °C). By using a W_1_/O/W_2_ technique, g-CA-PLGA improved the encapsulation efficiency (*EE*), suggesting that the presence of caffeic residues improved the compatibility between components (*EE*_PLGA_ = 35.0% ± 0.7% *vs.* *EE*_g-CA-PLGA_ = 95.6% ± 2.7%). Microspheres particle size distribution ranged from 15 to 50 µm. The zeta-potential values of placebo and loaded microspheres were −25 mV and −15 mV, respectively. The irradiation of g-CA-PLGA at the dose of 25 kGy caused a less than 1% variation of *M_w_* and the degradation patterns of the non-irradiated and irradiated microspheres were superimposable. The OVA content in g-CA-PLGA microspheres decreased to a lower extent with respect to PLGA microspheres. These results suggest that g-CA-PLGA is a promising biodegradable material to microencapsulate biological drugs.

## 1. Introduction

Poly(lactide-co-glycolide) (PLGA) is one of the most exploited polymer to design drug delivery systems and medical devices intended for parenteral use due to its excellent biocompatibility and ability to control drug release over a prolonged period of time. However, it was demonstrated that the end-sterilization by high-energy ionizations may alter the stability of the polymer [1,2,3,4] and the drug [5,6,7] as well as the release kinetics of encapsulated drugs [8]. Hence, measures have to be taken to balance the requirement of sterility assurance and the changing fundamental properties of a drug delivery system after irradiation.

Several strategies were developed in the attempt to avoid or minimize the detrimental effects of ionizing radiation on PLGA drug delivery systems. For instance, it was demonstrated that PLGA should be preferentially irradiated in inert atmosphere [2,3] or at low temperature [9]. In the case of vaccine, one approach to avoid detrimental effect on immunogenicity was to irradiate empty PLGA microspheres and in a second step, to adsorb sterile antigens on the particle surface [10]. Alternatively, the introduction of a radio-protecting ingredient has been also proposed. For instance, it was demonstrated that polyethylene glycol was able to preserve integrity of ovalbumin during the sterilization by irradiation due to a perturbation of the formation of perthiyl radicals [7]. Moreover, the use of hydroxypropyl methylcellulose as stabilizer during the microsphere preparations reduced the detrimental effect of β-irradiation on morphology, chemical, and physicochemical properties of methylprednisolone loaded microspheres [11].

More recently, anti-oxidants grafted onto the backbone of PLGA were proposed as novel biodegradable materials stable to gamma irradiation at a 25 kGy dose since the sterilization treatment caused only a very slight decrease of their molecular weight and the degradation patterns of the non-irradiated and irradiated material were superimposable [12]. The value of the proposed strategy consists in the protection of the PLGA from ionizing radiation without affecting the efficacy of the sterilization process. As a matter of fact, the European Pharmacopeia states the terminal sterilization is assured by a dose of 25 kGy. This implies that this irradiation dose permits to obtain a sterility assurance level higher than 10^−6^ independently of the initial bioburden and product characteristics.

Nevertheless, no information on the feasibility to encapsulate biological drugs in anxi-oxidant grafted PLGA has been made available.

In the current work, we compared the ability of caffeic acid grafted PLGA (g-CA-PLGA) to microencapsulate ovalbumin (OVA) with respect to the corresponding PLGA. OVA was selected as model protein because of its important role as adjuvant in the development of vaccines [13] and its sensitiveness of γ-radiation [7].

## 2. Results and Discussion

### 2.1. Chemical and Physico-Chemical Characterization of g-CA-PLGA

g-CA-PLGA conjugate was synthesized by a free radical grafting procedure involving the use of ascorbic acid/hydrogen peroxide redox pair as water-soluble and biocompatible initiator system. Compared to conventional initiator systems, indeed, the employed redox pair allows the performance of the reaction at lower temperatures reducing the risks of caffeic acid (CA) degradation and, at the same time, avoiding the formation of toxic side products.

The adopted synthetic strategy involves two main steps. The former involves the generation of hydroxyl radicals due to the oxidation of ascorbic acid by hydrogen peroxide at room temperature. These reactive species activate PLGA chain toward radical reactions promoting the insertion of antioxidant molecules onto the polymeric backbone. In the latter, the preformed macroradicals react with CA molecules inducing a PLGA-antioxidant covalent bond. This second step is favored since these reactions are characterized by a statistical behavior. The possibility that two macroradicals (macromolecules) can interact to form a new bond is very low. Instead, it is quite probable that in our conditions (concentration of reactants, solvent and temperature) a small molecule (CA) can move easily and react with PLGA. In any case, some side reactions occur and affect the polymeric backbones decreasing the *M_n_* value and consequently increase the PI value (Table 1). On the other hand, the adopted synthetic strategy did not significantly affect the distribution of polymer *M_w_* and this means that most of the macromolecules are preserved from side reactions. This was also confirmed by the glass transition values before and after reaction, that are very close (Table 1).

**Table 1:** Chemical and physico-chemical properties of poly(lactide-co-glycolide) (PLGA) and caffeic acid grafted (g-CA)-PLGA.

Polymer	*M_w_* (kDa)	*M_n_* (kDa)	PI	*T_g_* (°C)	DPPH inhibition (%)			
					1 h	2 h	3 h	24 h
PLGA	26.3 ± 1.3	17.3 ± 1.5	1.5 ± 0.0	47.1 ± 0.5	0 ± 0.3	0 ± 0.3	0 ± 0.5	0 ± 0.4
g-CA-PLGA	22.9 ± 0.7	8.4 ± 0.4	2.7 ± 0.0	45.0 ± 0.7	92 ± 0.6	93 ± 0.4	93 ± 0.5	98 ± 0.3

**Figure 1:**
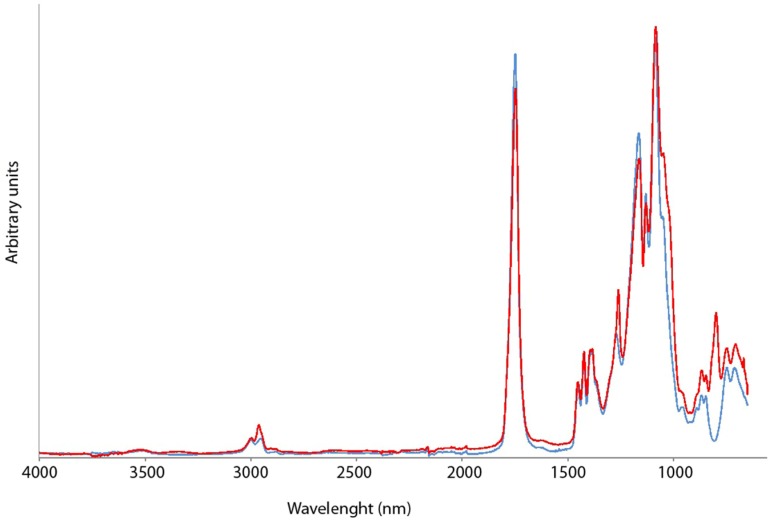
ATR-FTIR spectra of PLGA (blue line) and g-CA-PLGA (red line).

ATR-FTIR studies were carried out in three different regions, namely the aliphatic C–H stretching region (3000 and 2850 cm^−1^), the C=O stretching bands (1850 and 1650 cm^−1^) and the asymmetric stretching vibration at (1300–1000 cm^−1^) (Figure 1). The spectra evaluation was mainly interpreted in the region of carbonyl and the asymmetric stretching vibrations as the bond to CA had a significant effect on the bands at 1746 cm^−1^ and 1270 cm^−1^ which in g-CA-PLGA shifted to 1749 cm^−1^ and 1260 cm^−1^, respectively. Moreover, the appearance of the peak at 979 cm^−1^ was considered diagnostic of the grafting (Figure 1).

The antioxidant properties of the synthesized g-CA-PLGA conjugate were investigated by performing the DPPH assay. The DPPH radical is a stable organic free radical with an absorption maximum band around 515–528 nm and, therefore, it is a useful reagent for the evaluation of the antioxidant properties of compounds. In the performed assay, the antioxidant molecules reduce the DPPH radical to a yellow-colored compound, *i.e.*, diphenylpicrylhydrazine, and the extent of the reaction depends on the hydrogen donating ability of antioxidants. As reported in Table 1, the obtained results highlighted the good scavenging properties of PLGA conjugates, while the control polymer did not show any activities.

Since the antioxidant activity of the prepared conjugate is derived from phenolic groups present onto the polymeric chain, it is useful to express the antioxidant potential in terms of phenolic content. For this purpose, the Folin-Ciocalteu phenol reagent was used to obtain a rough estimation of the amount of disposable phenolic groups. Phenolic compounds, indeed, undergo a complex redox reaction with phosphotungstic and phosphomolybdic acids present in the Folin-Ciocalteu reactant. The color development is due to the transfer of electrons at basic pH to reduce the phosphomolybdic/phosphotungstic acid complexes to form chromogens in which the metals have lower valence. The results were expressed as mg equivalent of CA by comparing the obtained data to the CA calibration curve. In particular, for g-CA-PLGA this value was equal to 2.16 ± 0.7 mg/g of dry polymer.

### 2.2. OVA Loaded Microspheres

In the case of PLGA, the geometry of the devices has a strong influence on the bulk erosion profile. For instance, Witt and Kissel observed that in the case of microspheres, the high surface to volume ratio led to a shorter onset time of erosion; meanwhile in the case of rods a bulk erosion kinetic was observed [14]. Hence, the evaluation of the effect of γ-irradiation at the dose of 25 kGy was carried out directly on microspheres.

The encapsulation efficiency of OVA in the case of PLGA resulted a half of the theoretical drug content; in the case of g-CA-PLGA it was almost complete (Table 2) suggesting that the presence of CA residues improved the compatibility between the copolymer and the protein.

Since the process conditions were mild, the manufacturing of microspheres and the molecular weights of both polymers were not affected by the preparation process (*p* > 0.05).

**Table 2:** Physicochemical properties of placebo and ovolbumin (OVA) loaded microspheres.

Polymer	*EE*%	Particle Size (µm)			Span	ζ (mV)
		*d*_10_	*d*_50_	*d*_90_		
PLGA	–	6.4 ± 0.8	13.8 ± 0.0	38.5 ± 0.0	2.3 ± 0.1	−29.8 ± 1.3
g-CA-PLGA	–	5.8 ± 0.0	13.8 ± 1.1	40.7 ± 13.7	2.5 ± 0.8	−24.8 ± 1.4
PLGA	35.0 ± 0.7	5.5 ± 1.2	13.1 ± 0.9	29.4 ± 8.2	1.8 ± 0.3	−11.2 ± 3.0
g-CA-PLGA	95.6 ± 2.7	4.5 ± 0.1	15.4 ± 1.3	40.45 ± 1.1	2.3 ± 0.7	−15.1 ± 1.1

Particle size distribution of microspheres was in the range 15–50 µm. The grafting of CA led to prepare larger microspheres, using the same experimental set-up (Table 2). This could be explained by a different ability to coordinate the water molecules due to the presence of caffeic residues and, therefore, an increase in hydration of the polymer matrix.

Key factors defining the acceptability of a PLGA injectable suspension include syringeability and injectability [15,16]. Electrical charge density (zeta-potential) on the surface of microspheres is a critical aspect determining the injectability of the freeze-dried microspheres when reconstituted before injection. As the absolute value of electrical charge density increases, the microspheres tend to acquire more intensive repulsive forces, which prevent contact between them. Generally speaking, zeta-potential values of about ±20–30 mV allow obtaining a stable suspension of PLGA microspheres avoiding the formation of aggregates which can clog the needle.

Placebo microspheres made of PLGA exhibited a negative zeta-potential of about −30 mV because of the carboxylic end groups of PLGA (Table 2). The use of the chemically modified polymer to prepare placebo microspheres increased the zeta-potential to about −25 mV (Table 2). When OVA was loaded into both types of microspheres, a sharp reduction of zeta-potential was observed because of the protein absorption on the surface (Table 2). As a consequence, appropriate excipients, e.g., polysorbate 80, mannitol or carboxymethyl cellulose sodium [17], should be added to the solvent to facilitate the reconstitution of the suspension before injection

Drug release and drug release rate from PLGA-based controlled release systems is a rather complex process influenced by several steps, e.g., diffusion of water into the system, drug dissolution, drug diffusion out of the delivery system, polymer swelling and matrix erosion [18]. In particular, diffusion and degradation/erosion of the polymer are considered the two main mechanisms governing the drug release. In the early stage, the release rate is considered to be diffusion-controlled which is critical for macromolecules due to their large molecular volume. Thus, in the present study, we determined the OVA release over the first week of incubation in physiological medium.

**Figure 2:**
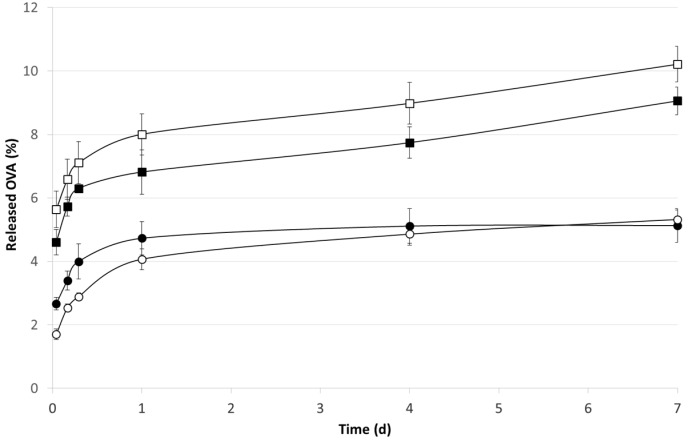
*In vitro* release pattern of OVA from PLGA (circle) and g-CA-PLGA (square) before (black symbol) and after γ-irradiation (empty symbol) at the dose of 25 kGy. The results are expressed as the mean of three determinations ± error standard.

Different patterns were evident on the basis of the polymer type (Figure 2). In the case of PLGA microspheres, OVA was detectable only in the first 24 h, indicating that only the protein adsorbed on the polymeric structure was released in agreement with the literature data [8,19]. The presence of OVA on the microsphere surface was also in agreement with the strong reduction of the zeta potential registered for PLGA microspheres.

**Figure 3:**
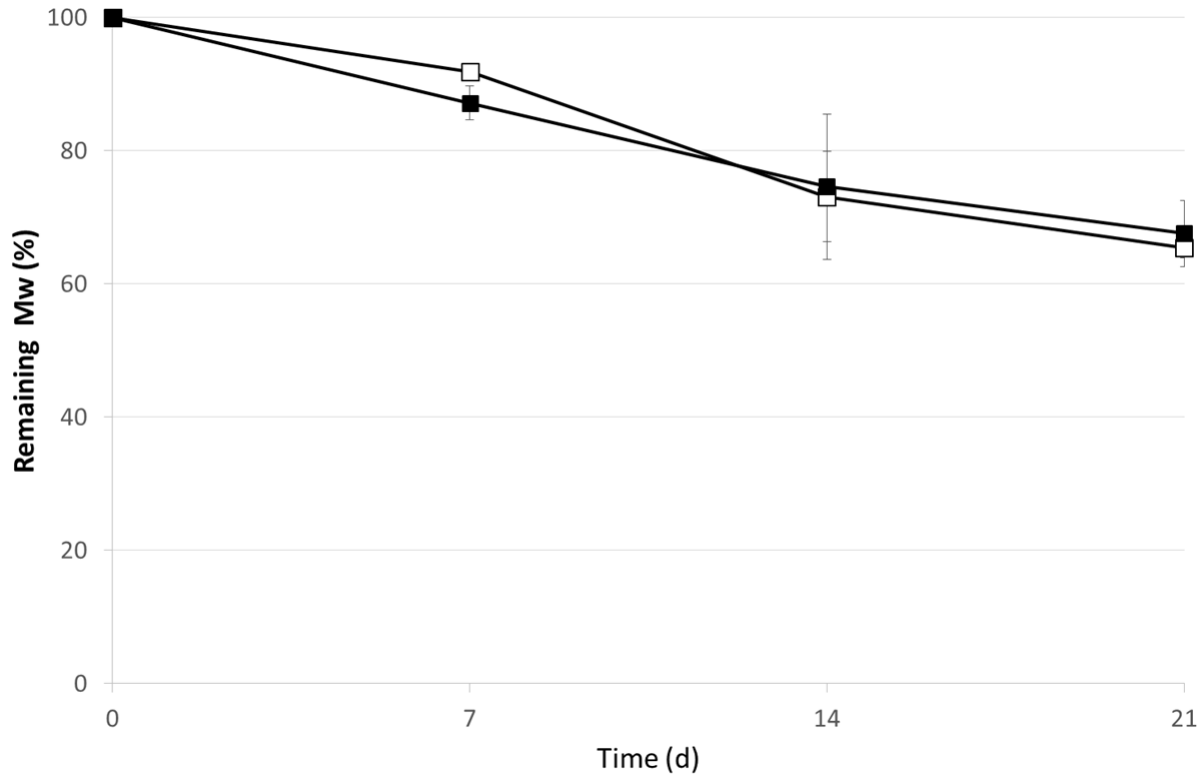
*In vitro* degradation pattern of g-CA-PLGA microspheres before (black symbol) and after γ-irradiation at the dose of 25 kGy (empty symbol). The results are expressed as the mean of three determinations ± standard deviation.

Drug release rates from g-CA-PLGA microspheres were improved resulting in more continuous release profiles by contrast to PLGA microspheres. Indeed, the microspheres prepared by using g-CA-PLGA showed a linear relationship between the OVA released fraction and the square root of time indicating that the drug release was controlled only by a diffusion process (*R*^2^ = 0.97). Indeed, no significant variations in the polymer *M_w_* occurred up to 14 days (Figure 3). This peculiar pattern might cause also the slight increase in burst effect with respect to that determined in the case of PLGA microspheres.

It can be assumed that the presence of CA on PLGA backbone reduced the interpolymeric entanglement as demonstrated by the reduction of the *T_g_* values and by the shift of the C=O and OH stretching vibrations towards lower wavenumbers.

2.3. γ-Irradiation of Microspheres

After irradiation a significant decrease on PLGA *M_w_* form 26.0 ± 1.1 to 21.6 ± 1.3 was noticed along with an increase of PI from 1.6 ± 0.0 to 2.1 ± 0.1. The grafting of CA on the PLGA backbone determined a strong increase of the polymer resistance to γ-rays since only the *M_w_* decreased from 22.5 ± 0.8 to 22.0 ± 1.0 kDa, meanwhile the PI did not undergo to significant changes [PI before irradiation = 2.7 ± 0.0 (Table 1); PI after irradiation = 2.7 ± 0.2]. These data on g-CA-PLGA were in agreement with previously results on pyrogallic acid covalently bonded on PLGA [12] confirming the suitability of this approach to design a novel biomaterials stable to final sterilization by ionizing radiation in overkill conditions [20].

**Figure 4:**
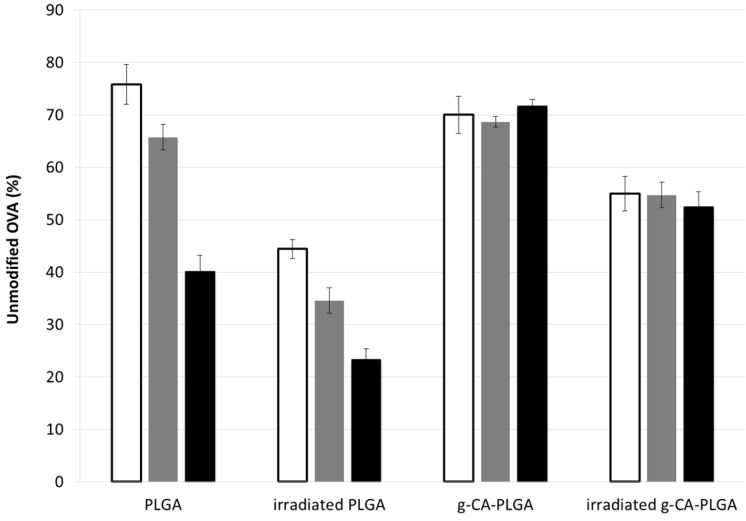
Percentage of unmodified OVA released from PLGA and g-CA-PLGA microspheres before and after γ-irradiation at the dose of 25 kGy after 1 (white bar), 2 (gray bar) and 6 h (black bar) during the *in vitro* release test in physiologic medium.

After irradiation, the total amount of OVA released from PLGA microspheres appeared influenced by the treatment in a very low extent and only at the early stage (Figure 3). However, since the percentage of unmodified OVA (*i.e.*, the monomer determined by SEC) was significantly reduced of about 45% halved (time point: 1 h, *p* < 0.01) with respect to the not-irradiated microspheres (Figure 4), the decrease might be attributed to an increase of protein dimension, namely formation of dimers and/or multimers, mediated by the formation of the perthiyl radicals on cysteine residues [7].

In contrast, the total amount of OVA released from g-CA-PLGA microspheres was not affected by the irradiation treatment. Indeed, no differences were detected between the two release patterns as evidenced by the standard error depicted in Figure 3 and the *p*-values calculated at each individual point (0.36 < *p* < 0.87). Nevertheless, as depicted in Figure 4 after 1 h, the amount of unmodified OVA released decreased of about 15% (*p* = 0.04). The differences and peculiarities of the release patterns found in the two cases can be attributed to a an alteration of the structure of both polymeric carrier, taking place via H abstraction reactions [4] and OVA, which occurred mainly via perthiyl formation [7]. However, the scavenger properties of CA probably reduced the extent of the radiolytic yields and this can justify the reduced alterations of g-CA-PLGA formulation due to the irradiation process [21,22]. Nevertheless, to confirm this hypothesis and determine the radiolytic mechanisms, a specific EPR study, which is beyond the aim of this work, should be performed.

## 3. Experimental Section

### 3.1. Synthesis of g-CA-PLGA

An amount of 0.5 g PLGA (lactide/glycolide mole ratio: 53/47) was dissolved in 5 mL of tetrahydrofuran (THF). The resulting solution was deposited as a thin film in a round bottom flask after THF evaporation in a vacuum pump for 3–8 h at room temperature. Then, 50 mL of a 1.0 M H_2_O_2_ solution containing 1.2 g of ascorbic acid were added.

After 30 min, the H_2_O_2_ solution was removed and replaced with 50 mL of H_2_O_2_ (2.0 M)/ethanol mixture (1:1 v/v) containing 1.2 g of ascorbic acid and 0.8 mmol of caffeic acid (CA). The mixture was maintained at 25 °C for 24 h under atmospheric air.

At the end of reaction, the functionalized film was freed from solvent, exhaustively washed with ethanol and distilled water, in order to remove the unreacted CA, and finally dried under vacuum for 24 h at room temperature.

### 3.2. Characterization of g-CA-PLGA

#### 3.2.1. Determination of Scavenging Activity on DPPH Radicals

In order to evaluate the free radical scavenging properties of the synthesized conjugate, the reactivity towards a stable free radical, 2,2′-diphenyl-1-picrylhydrazyl radical (DPPH), was evaluated [12]. Samples of g-CA-PLGA or PLGA weighting 25 mg were added to 25 mL of an acetonitrile solution of DPPH (100 μM) and incubated in a water bath at 25 °C. After 1, 2, 3 and 24 h, the absorbance of the remaining DPPH was determined colorimetrically at 517 nm. The same reaction conditions were applied to the control polymer to evaluate the interference of the polymeric material on DPPH assay. The scavenging activity of the tested polymers was expressed as percent inhibition of DPPH radicals and calculated according to the following Equation (1):

(1) $$inhibition\%=\frac{A_{0}-A_{1}}{A_{0}}\times 100$$

where *A*_0_ is the absorbance of a standard prepared in the same conditions, but without any polymers, and *A*_1_ is the absorbance of the polymeric samples. Each measurement was carried out in triplicate and data expressed as means (±SEM).

#### 3.2.2. Evaluation of the Disposable Phenolic Groups by Folin-Ciocalteu Assay

A sample of about 30 mg of g-CA-PLGA was dispersed in 6 mL of distilled water in a volumetric flask. Folin-Ciocalteu reagent (1 mL) was added and the content of the flask was mixed thoroughly. After 3 min, 3 mL of a Na_2_CO_3_ solution (2% w/v) were added and, then, the mixture was allowed to stand for 2 h with intermittent shaking. The absorbance was measured at 760 nm against a control prepared using the blank polymer under the same reaction conditions.

The amount of total phenolic groups was expressed as CA equivalent concentrations by using the equation obtained from the calibration curve of the antioxidant molecule. This one was recorded by employing five different CA standard solutions. Aliquots (0.5 mL) of each solution were added to the Folin-Ciocalteu system to obtain the final concentration of 8.0, 16.0, 24.0, 32.0, and 40.0 μM, respectively. After 2 h, the absorbance of the solutions was measured to record the calibration curve and the correlation coefficient, slope and intercept of the obtained regression equation were calculated by the method of least square.

### 3.3. Molecular Characterization

#### 3.3.1. ATR-FTIR Spectroscopy

About 2.0 mg sample was place on a diamond crystal mounted in ATR cell (Perkin Elmer, Monza, Italy). FTIR measurements were performed with Spectrum™ One spectrophotometer (Perkin Elmer, Monza, Italy). The spectra were recorded at 4 cm^−1^ resolution and 128 scans were collected over the wavenumber region 4000–650 cm^−1^.

#### 3.3.2. Thermal Analysis

DSC data were recorded by using a DSC 1 Stare System (Mettler Toledo, Novate Milanese, Italy). Samples of about 2 mg exactly weighted (±0.01 mg) were sealed in pin holed aluminum pans and subject to two cooling and heating cycles from 25 to 100 °C at the cooling and heating rate of 20 K·min^−1^. The DSC cell and RCS were purged with dry nitrogen at 80 and 120 mL/min, respectively. Glass transition temperature (*T_g_*) was measured on the second heating ramp and the results are expressed as the mean of two determinations.

#### 3.3.3. GPC Analysis

Polymer molecular weights were determined by using a HP1100 Chemstation (Agilent, Santa Clara, CA, USA) equipped with a combination of two columns: µStyragel™ Toluene 104 Å 7.8 × 300 mm and µStyragel™ Toluene 103 Å 7.8 × 300 mm (Waters, Milan, Italy). Chromatographic conditions. Mobile phase: THF; flow rate: 1.0 mL·min^−1^; detector: refractive index signal; injection volume: 20 µL. The molecular weight (*M_w_*) of each sample was calculated using a calibration curve made with monodisperse polystyrene standards, *M_w_* ranging from 1000 to 45,000 Da.

### 3.4. Placebo and Ovalbumin Loaded Microspheres

#### 3.4.1. Microspheres Preparation

Placebo and ovalbumin (OVA) loaded microspheres were prepared by a water-in-oil-in-water (W_1_/O/W_2_) emulsion/solvent evaporation method using HPMC as emulsifier [11]. Briefly, OVA (10% w/w theoretical drug loading) was dispersed in a 20% w/w PLGA or g-CA-PLGA solution in dichloromethane. After mixing by vortex and sonicating, the W_1_/O suspension was slowly injected into 25 mL of 2.5% HPMC solution under stirring at 600 rpm at 5 ± 3 °C. The resulting W_1_/O/W_2_ system was then poured into 350 mL water and stirred at 600 rpm and 40 °C for 3 h for microsphere hardening. The resulting microspheres were filtered by a 1.2 µm nitrocellulose filter (Millipore, Milan, Italy), washed with MilliQ^®^ water for 15 min in order to eliminate the protein adsorbed on the surface of microspheres and freeze-dried (Freeze-drying system Alpha 1. Martin Christ, Osterode am Harz, Germany). After preparation, the microspheres were stored at 4 °C until use.

#### 3.4.2. γ-Irradiation

OVA, placebo and drug loaded microspheres were irradiated by using ^60^Co as irradiation source (Gammarad Italia S.p.A., Bologna, Italy) in the presence of air at 25 kGy dose (dose rate = 516 Gy/h) and 25 °C.

#### 3.4.3. Microspheres Characterization

##### Particle Size

An Accusizer 770 (PSS Inc., Port Richey, FL, USA) using the technique “Single Particle Optical Sensing” was used to determine the size distribution of microspheres. About 2 mg of lyophilized microspheres exactly weighed were suspended into a 0.02% polysorbate 80 aqueous solution to prevent aggregation [23]. Particle size was expressed as undersize cumulative percentages and the population dispersity was referred as span and calculated as reported in the Equation (2):

(2) $$span=\frac{d_{90}-d_{10}}{d_{50}}$$

where *d*_90_, *d*_10_, and *d*_50_ are the mean diameters at the 90%, 10% and 50% of the population distribution, respectively.

##### Zeta-Potential

The particle charge was quantified as the zeta potential by laser Doppler velocimetry and phase analysis light scattering. Zeta potential measurements were carried out in phosphate buffer at pH 7.4 (ionic strength = 0.01) by using a Zetasizer nano ZS (Malvern Instruments, Malvern, UK). The results are shown as means ± standard deviation (*n* = 3).

##### Total Protein Content Determination

The total OVA content was determined after microsphere digestion through alkaline sodium dodecil sulphate (SDS) solution, as previously described [7]. The total amount of protein was determined with a bicinchoninic acid (BCA) protein assay reagent kit (Thermo Scientific, Milan, Italy), by colorimetric analysis at 562 nm with a UV spectrophotometer Beckman DU640 (Beckman Coulter, Pasadena, CA, USA). Three replicates of all samples and standards were assayed. Encapsulation efficiency (*EE*%) was expressed as the percentage of protein entrapped compared to theoretical drug content. Theoretical drug content was defined as the ratio between the amount of protein added to the formulation over the total amount of polymer plus protein, while actual loading was ratio between the amount of entrapped protein as determined by BCA assay and the microsphere batch mass.

### 3.5. In Vitro OVA Release

The amount of OVA released from microspheres was evaluated in bottles closed by screwed stoppers and stirred in a shaker incubator (60 strokes/min) at 37 ± 2 °C. Samples of microspheres exactly weighed (±0.01 mg) were suspended in 2 mL of dissolution medium consisting in pH 7.4 phosphate buffer saline. At determined time points, 2 mL dissolution medium was withdrawn and replaced with fresh buffer. The total amount of OVA released was tested by the BCA method. The results are expressed as a mean of three parallel experiments.

### 3.6. Ovalbumin Determination

The amount of protein released was determined by BCA assay described above. Moreover, for the determination of OVA, its dimers and multimers, a HP1100 Chemstation (Agilent, Santa Clara, CA, USA) equipped with column (TSK-GEL column, 30 cm × 7.5 mm, TOSOH Biosciences, Rivoli, Italy) was used. Chromatographic conditions. Mobile phase: pH 7.4 phosphate buffer solution containing 0.01% NaN_3_; flow rate: 1.0 mL·min^−1^; detector: UV at 280 nm; injection volume: 80 µL; temperature: 25 °C. A calibration curve was built up using OVA standards in the ranging from 0.1 to 5 µg/min.

## 4. Conclusions

The grafting of CA significantly improved the features of PLGA since the antioxidant allowed to ameliorate the encapsulation efficiency of OVA and permitted a continuous diffusion of the protein in the early stage of drug release. Furthermore, the anti-oxidant properties prevent both the polymer degradation and the OVA dimerization process driven by radical pattern suggesting the feasibility of a final sterilization by ionizing radiation.

All together, these data indicate that g-CA-PLGA is a promising biomaterial to design biodegradable microspheres for the parenteral delivery of biological drug.

## References

[1] Hausberger A.G., Kenly R.A., DeLuca P.P. (1995). Gamma irradiation effects on molecular weight and *in vitro* degradation of poly(lactide-co-glycolide) microparticles. Pharm. Res..

[2] Montanari L., Constantini E.C., Signoretti L., Valvo L., Santucci M., Bartolomei P., Fattibene P., Onori S., Faucitano A., Conti B., Genta I. (1998). Gamma irradiation effects on poly(d,l-lactide-co-glycolide) microspheres. J. Control. Release.

[3] Montanari L., Cilurzo F., Valvo L., Faucitano A., Buttafava A., Groppo A., Genta I., Conti B. (2001). Gamma irradiation effects on stability of poly(lactide-co-glycolide) microspheres containing clonazepam. J. Control. Release.

[4] Faucitano A., Buttafava A., Montanari L., Cilurzo F., Conti B., Genta I., Valvo L. (2003). Radiation-induced free radical reactions in polymer/drug systems for controlled release: An EPR investigation. Rad. Phys. Chem..

[5] Montanari L., Cilurzo F., Conti B., Genta I., Groppo A., Valvo L., Faucitano A., Buttafava A. (2002). Gamma irradiation effects and EPR investigation on poly(lactide-co-glycolide) microspheres containing bupivacaine. Farmaco.

[6] Mohr D., Wolff M., Kissel T. (1999). Gamma irradiation for terminal sterilization of 17β-estradiol loaded poly(d,l-lactide-co-glycolide) microparticles. J. Control. Release.

[7] Dorati R., Genta I., Montanari L., Cilurzo F., Buttafava A., Faucitano A., Conti B. (2005). The effect of γ-irradiation on PLGA/PEG microspheres containing ovalbumin. J. Control. Release.

[8] Montanari L., Cilurzo F., Selmin F., Conti B., Genta I., Poletti G., Orsini F., Valvo L. (2003). Poly(lactide-co-glycolide) microspheres containing bupivacaine: Comparison between gamma and beta irradiation effects. J. Control. Release.

[9] Fernandez-Carballido A., Puebla P., Herrero-Vanrell R., Pastoriza P. (2006). Radiosterilisation of indomethacin PLGA/PEG-derivative microspheres: Protective effects of low temperature during gamma-irradiation. Int. J. Pharm..

[10] Jain S., Malyala P., Palloro M., Giuliani M., Petersen H., O’Hagan D.T., Singh M. (2011). A two-stage strategy for sterilization of poly(lactide-co-glycolide) particles by gamma-irradiation does not impair their potency for vaccine delivery. J. Pharm. Sci..

[11] Cilurzo F., Selmin F., Minghetti P., Montanari L. (2008). Design of methylprednisolone biodegradable microspheres intended for intra-articular administration. AAPS PharmSciTech..

[12] Cilurzo F., Puoci F., Selmin F., Iemma F., Minghetti P. (2011). Pyrogallic acid-PLGA conjugate as new biodegradable material suitable for final sterilization by irradiation. Pol. Adv. Tech..

[13] Mohanan D., Gander B., Kündig T.M., Johansen P. (2012). Encapsulation of antigen in poly(d,l-lactide-co-glycolde) microspheres protects from harmful effects of γ-irradiation as assessed in mice. Eur. J. Pharm. Biopharm..

[14] Witt C., Kissel T. (2001). Morphological characterization of microspheres, films and implants prepared from poly(lactide-co-glycolide) and ABA triblock copolymers: Is the erosion controlled by degradation, swelling or diffusion?. Eur. J. Pharm. Biopharm..

[15] Floyd A.G., Lieberman H.A., Rieger M.M., Banker G.S. (1996). Injectable emulsions and suspensions. Pharmaceutical Dosage Forms—Disperse Systems.

[16] Cilurzo F., Selmin F., Minghetti P., Adami M., Bertoni E., Lauria S., Montanari L. (2011). Injectability: An open issue. AAPS PharmSciTech..

[17] Panusa A., Selmin F., Rossoni G., Carini M., Cilurzo F., Aldini G. (2011). Methylprednisolone-loaded PLGA microspheres: A new formulation for sustained release via intra-articular administration. A comparison study with methylprednisolone acetate in rats. J. Pharm. Sci..

[18] Fredenberg S., Wahlgren M., Reslow M., Axelsson A. (2011). The mechanisms of drug release in poly(lactic-co-glycolic acid)-based drug delivery systems—A review. Int J. Pharm..

[19] Conti B., Groppo A., Genta I., Dacarro C., Valvo L., Cilurzo F., Montanari L. Comparison among different preparation methods of ovalbumin loaded PLGA microspheres. Proceedings of 27th International Symposium on Controlled Release of Bioactive Materials.

[20] (1991). The Use of Ionizing Radiation in the Manufacture of Medicinal Products.

[21] Audette-Stuart M., Houée-Levin C., Potier M. (2005). Radiation-induced protein fragmentation and inactivation in liquid and solid aqueous solutions. Role of OH and electrons. Radiat. Phys. Chem..

[22] Terryn H., Deridder V., Sicard-Roselli C., Tilquin B., Houée-Levin C. (2005). Radiolysis of proteins in the solid state: An approach by EPR and product analysis. J. Synchrotron Radiat..

[23] Selmin F., Blasi P., DeLuca P.P. (2012). Accelerated polymer biodegradation of risperidone poly(d,l-lactide-co-glycolide) microspheres. AAPS PharmSciTech..

